# Exploring primary care access in Finland: a mixed-methods study in the context of the updated waiting-time guarantee

**DOI:** 10.1093/eurpub/ckag121

**Published:** 2026-07-14

**Authors:** Markku Satokangas, Laura Kihlström, Satu Paatela, Ilmo Keskimäki, Henna Paananen, Liina-Kaisa Tynkkynen

**Affiliations:** Welfare State Research, Finnish Institute for Health and Welfare, Helsinki, Finland; Advanced Care Research Centre, Usher Institute, University of Edinburgh, Edinburgh, United Kingdom; Welfare State Research, Finnish Institute for Health and Welfare, Helsinki, Finland; Welfare State Research, Finnish Institute for Health and Welfare, Helsinki, Finland; Welfare State Research, Finnish Institute for Health and Welfare, Helsinki, Finland; Faculty of Social Sciences, Tampere University, Tampere, Finland; Faculty of Management and Business, Tampere University, Tampere, Finland; Welfare State Research, Finnish Institute for Health and Welfare, Helsinki, Finland

## Abstract

Prompt primary care (PC) access is salient for equitable care. Finland has a high level of unmet health needs, characterized by stark socioeconomic disparities and accounted for by long waiting times. In September 2023, Finland updated its legislation regulating waiting times to non-urgent general practitioner (GP) consultations, reducing the maximum waiting time to 14 days. Hereafter, PC access statistics improved. We assessed patients’ views towards PC access after the policy update and how they reflect against national statistics. This is an exploratory sequential mixed-methods study in rural North Karelia, Finland. Interviews with patients and staff (*n* = 40) and observations (150+ hours) were conducted in a public PC centre in January–March 2024. Data on GP consultations including waiting times were obtained annually from individual-level national registers in 2017–24. Older patients with chronic health needs stated increased difficulties in navigating an increasingly digital PC and lack of access to in-person consultations. Staff reported an accelerated need for ‘gatekeeping’ access to GP consultations due to lack of resources. The number of all GP consultations increased 1.69-fold from 2017 to 2024, but in-person ones reduced 0.60-fold. A change in definition explained the improvement of waiting time statistics. A more robust waiting-time guarantee in Finland took place in tandem with austerity measures and a rapid shift to telemedicine. Improved PC access statistics conflict with local experiences and ignore reduced provision of in-person GP consultations, thus hiding the associated equity impacts. Our results call for investing in more nuanced monitoring of policy changes.

## Introduction

Prompt and reliable access to healthcare is a key determinant of population health [1]. Primary care (PC) is often conceptualized as the backbone of healthcare, given that its access associates with a ‘more equitable distribution of health in populations’ [2, 3]. Moreover, PC access is one of the key indicators against which health systems are evaluated, such as through EU-wide self-reported data on unmet care needs based on difficulties in accessing healthcare because of financial reasons, timeliness (waiting lists), or distance/transport [4]. In parallel to realized PC utilization, register data can provide information on, e.g. waiting times from booking to general practitioner (GP) consultation as well as potential proxies for poor PC access, such as self-referred emergency department (ED) visits [5].

PC access data are salient for health system performance assessment and policymaking. However, how access is defined and measured has been critiqued from various viewpoints, notably through the observation that focusing on access solely through timeliness or affordability does not sufficiently capture patient or health workforce realities and preferences [6, 7]. Additionally, a tendency to report access at the population-average level may mask critical inequities [8]. All this calls for research which can enrich the ways in which PC access is measured and reported.

This study contributes to these methodological considerations regarding PC access by examining the impacts of the Finnish waiting-time guarantee legislation update [9]. Finland has a tax-funded public PC which provides universal access but struggles with excess waiting times to non-urgent GP consultations [10, 11]. The Finnish Health care act (1326/2010) obliges public PC to ensure that patients can contact them during weekday office hours—either by phone, digitally or face-to-face—and receive a same day assessment of their care needs, which also initiates the calculation of waiting times. This assessment is done by nurses who consequently spend much time responding to phone calls and digital inquiries and act as gatekeepers to GP consultations. To ease up the demand for GP consultations, public PC has also transferred several responsibilities from GPs to nurses, access to whom is good throughout Finland. This broad role of nurses is reflected in Finland having fewer physicians and more nurses than the EU average [10]. Moreover, since the COVID-19 pandemic, public PC has increasingly shifted to providing GP consultations as remote ones. Alongside public PC, Finland has alternative pathways to GP consultations, of which especially private healthcare and occupational healthcare compete with public PC for workforce and often provide same day GP access for the affluent population and employees [12, 13]. It can thus be stated that over time the Finnish PC has developed into a polarized system with structural inequities for accessing GP care. The update of waiting-time guarantee legislation signed into law in January 2023 sought to reduce patients maximum waiting times in public PC (Table 1), heeding to longstanding calls to strengthen public PC access.

**Table 1.** Brief summary of PC waiting-time guarantee legislation in Finland

Description	Introduced	Implemented
PC waiting time guarantee introduced		
First GP visit in new health problem within reasonable time, at latest within 3 months	September 2004	March 2005
Finnish Health Care Act amended (by a left-wing cabinet)		
PC waiting-time guarantee 14 days	January 2023	September 2023
PC waiting-time guarantee 7 days		November 2024
Finnish Health Care Act amended (by a right-wing cabinet)		
Abandoning transition to 7-day PC waiting-time guarantee	December 2023	November 2024
Finnish Health Care Act amended		
PC waiting-time guarantee 14 days for those under 23 years of age, and 3 months for rest of the population	December 2024	January 2025

This mixed-methods study aimed to examine the patients’ lived experiences about PC access following this policy update and assess whether these experiences were supported by register-based analyses. A central tenet of this work is that examining healthcare systems benefits from integrating quantitative and qualitative approaches [14]. This study provides an example of how a health policy change in Finland impacted patients’ access, and can inform other countries where they should invest to avoid possible pitfalls when pursuing better PC access via legislative measures.

## Methods

This study followed an exploratory sequential mixed methods design: quantitative data were collected and analysed after qualitative data collection and analysis [15]. Ethnographic fieldwork was carried out in PC setting in the rural North Karelia Wellbeing Services County (WBSC), where the key challenges include population aging, high disease burden, long distances to care and GP shortages. The fieldwork focused on the perspectives and experiences of service users and health and care workforce. Insights from this qualitative phase were then complemented and corroborated by quantitative analysis on PC access in the same region [14]. Together, these two forms of data were instrumental in addressing key questions related to PC access in the context of a policy update: (i) What is happening from the perspective of service users and workforce, (ii) How are these findings confirmed or challenged by quantitative data on PC access, and what do they reveal about the validity of access data?

### Data sources

Qualitative data were collected in a primary healthcare centre (HCC) and consist of semi-structured interviews conducted with patients (*n* = 29) and health workforce (*n* = 11) as well as observations and informal interviews in common areas of the HCC (150+ hours). These data were collected from January 2024 to March 2024. A researcher spent a total of 20 days in the HCC. For the semi-structured interviews, the researcher used an interview guide which included a general set of themes related to access to care, quality of care, and healthcare reform. At the studied HCC, the majority of interview participants were over 66 years old (75%), women (65%), retired (84%), and had one or more chronic health conditions (84%).

The quantitative, individual-level, linked register data were obtained for Finns living in the North Karelia WBSC in 2017–24 (*n* = 162 312–165 348 annually). For these individuals, all GP consultations to public PC and their waiting times in 2017–24 were obtained from the Register for Primary Health Care Visits, and all ED visits from the Care Register for Health Care. Individual income and municipality of residence were obtained from the FOLK population dataset of Statistics Finland. Finally, the number of GPs per year was identified from the consultations.

### Data analysis

The qualitative data were analysed using both deductive and inductive elements. Interview transcripts were imported into ATLASti.9. Due to the breadth of the data, transcripts were first coded deductively, coding segments related to access to care. This led to a coding a total of 59 (patients) and 16 (health workforce) segments in the transcripts. The coded segments were exported into Microsoft Excel, where the segments were further coded inductively to assess what topics were relevant within the category of access to care. Following this process, we present emerging themes in the data related to access to care both from the perspective of patients and workforce.

For reasons of HCC anonymity, the quantitative data were analysed and reported for the whole population of North Karelia. This included descriptive analyses in which in-person GP consultations and their waiting times, remote GP consultations as well as the number of times GPs were consulted by nurses were examined over time. The waiting times were calculated both with the old definition of statistics in 2017–24 and with a new definition from September 2023 onwards. The estimate for full-time equivalent (FTE) GPs was calculated by summing up the number of days when individual GPs provided at least one consultation. These numbers were calculated separately for each GP, summed up for all GPs and finally divided by the average time that a public healthcare employee worked annually in 2017–24 (1453–1560 h divided by the average length of a GP working day 7 hours 47 minutes) [16]. For assessing the time trends in socioeconomic disparities in GP consultations, concentration indices (*C*s) were calculated for those aged 25 and over (*n* = 121 734–123 272) with tabulated age-standardized and sex-standardized rates in eight time points–using a Monte Carlo simulation with 10 000 iterations when calculating confidence intervals [17–19]. In the case of positive outcome (here GP consultations), *C* value of 0 represents absence of disparities and a value of −1 absolute disparities favouring those with lower incomes. Finally, as a sensitivity analysis, all the quantitative analyses were replicated in the population of the municipality served by HCC from which the qualitative data were collected. The analyses were performed with R release version 4.4.1 and using the linear regression method [20].

### Ethical permits

All analysis were completed in accordance with the Finnish data protection legislation and the ethical guidelines of the Finnish National Board on Research Integrity. The study protocol was approved by the Research Ethics Committee of the Finnish Institute for Health and Welfare (THL); decision numbers (THL/657/6.02.01/2023§929 and THL/4501/6.02.01/2023§953). The qualitative case study was further approved by the North Karelia WBSC. The permissions to use the register data were obtained from THL and Statistics Finland. Quantitative analyses used pseudonymized secondary data and according to the Finnish data protection legislation, the participants were not contacted.

## Results

### Qualitative results

We categorized our findings into barriers and facilitators of PC access, supported by illustrative quotes. Both patients and staff viewed these barriers as part of a broader trend of welfare state retrenchment, budget cuts, and fewer permanent GP positions in rural areas. While long-standing, these issues were reported to have intensified in recent years. Under barriers and facilitators, we list themes raised by patients and workforce. Each quote is labelled by participant type (P = patient, GP = general practitioner, RN = registered nurse, PN = practical nurse) and interview order.

#### Barriers to accessing care

##### Impact of legislative changes

The updated waiting-time guarantee law, requiring access to a GP within 14 days, was met with varying responses at the HCC. On the one hand, the legislative change was seen as diverting health workforce attention to the number of phone calls responded to rather than to the quality of care, which may have resulted in unmet care needs. On the other hand, it was reported that the HCC had been successful in meeting the legislative criteria of a patient receiving care within 14 days. However, clinicians also argued that this was due to raising the criteria for patients to receive a non-urgent GP consultation, as summarized by one GP: ‘*It is difficult to report numerically what is happening in practice. Because our statistics do look really good, almost everyone who needs a GP consultation makes it to a GP within two weeks. But after the law changed, in practice it means that we just elevated the threshold of getting a GP consultation.*’ *(GP11).*

##### GP shortages and gatekeeping

Patients reported difficulty accessing GP appointments due to regional shortages. Some were even advised to seek private care by health workforce, which was financially unfeasible for many: ‘*I don’t understand why they have told me to just try and access a private GP. I have a small pension, I cannot afford to visit a private GP too often…but the nurses have said this several times, to visit a private GP. They have no GP appointments, they have no GPs*’ *(P17).* Health workforce recognized the difficulty in accessing GP consultations as a timely issue. The issue was described as resulting from long-standing underinvestment in PC as well as lack of GPs in the region, which resulted in nurses having to act as gatekeepers for GP consultations in the care needs assessment which was primarily done over the phone. ‘*There is a mental load in this work, because you get a lot of negative feedback from patients. It can be a bit challenging to work as a nurse between the patient and the doctor. Because there are not many doctors, so you are a kind of gatekeeper there for who gets to see the doctor. And sometimes there are these ethical ponderings, and you wonder if you did the right thing*’. (RN1).

##### Shift to remote-first care

The shift to remote care (e.g. phone consultations, digital platforms) posed challenges, especially for older or less tech-savvy patients: ‘*Given the age distribution here, they shouldn’t push everything to be remote. Some people just can’t manage it*’*. (P13*). Remote GPs, working outside the region, further contributed to patients’ sense that access had become more difficult and also led to discontinuity of care: ‘*We now have remote GPs. You don’t even know where your consultation is coming from*’. *(P14).* One GP noted that pre-COVID reforms in the region had prioritized remote consultations which had reduced face-to-face care and affected both patient access and staff morale: ‘*GPs became consultants for nurses. The goal was to make the phone system work, not to see patients*’. (GP2). Similarly, registered nurses also recognized the risk in doing most of the care needs assessment remotely: “*The way we do it these days is that nurses evaluate on a computer which symptoms qualify for access. And their job is to–to put it bluntly–to block those who don’t need care*’. (RN10). Thus, the shift to remote-first care in the rural North Karelia was also perceived as profoundly changing professional roles and affecting the morale of health and care workforce.

#### Facilitators to accessing care

Patient experiences with PC access varied. While some reported barriers, others described smooth and positive interactions. One patient suggested that negative perceptions might stem from the nurses’ roles as gatekeepers to GP consultations: ‘*Because nurses often guide the patient, some may think they have the final say on care… I think it works fine*’*. (P*3*)*. Some patients who had not faced difficulties found it hard to understand others’ complaints: ‘*I’ve had surprisingly smooth access, even though others say they can’t get in. My issues haven’t been serious, so I’ve managed just fine*’*. (P*15*)*. Moreover, patients who understood how to navigate the system—especially remote services—reported fewer issues: ‘*If you need help, you just ask. It’s always been easy for me to understand the system. First contact is by phone*’*. (P*2*)*. Some also mentioned using private services when needed and financially feasible.

The nature of the health concern influenced access and satisfaction, as stated by one GP: ‘*…When it comes to musculoskeletal issues, since there aren’t really any quick fixes or easy snap-of-the-fingers solutions, I don’t feel I can impact them as much. It’s more about guiding the patient to do their own exercises, but the actual improvement is really up to them. And that’s where the frustration comes in*’*. (GP*11*).* In other words, conditions like cardiovascular risks were seen as more manageable within PC, while musculoskeletal issues were harder to treat effectively. Finally, the presence of a staffed patient office near the entrance positively impacted patient experiences. Informal conversations revealed that simply being able to ask for help, such as directions or basic guidance, improved patients’ moods and overall satisfaction with their visit. Yet, according to our observations, the patient office was no longer planned to be staffed in the future due to budget cuts.

### Quantitative results

**Figure 1.**
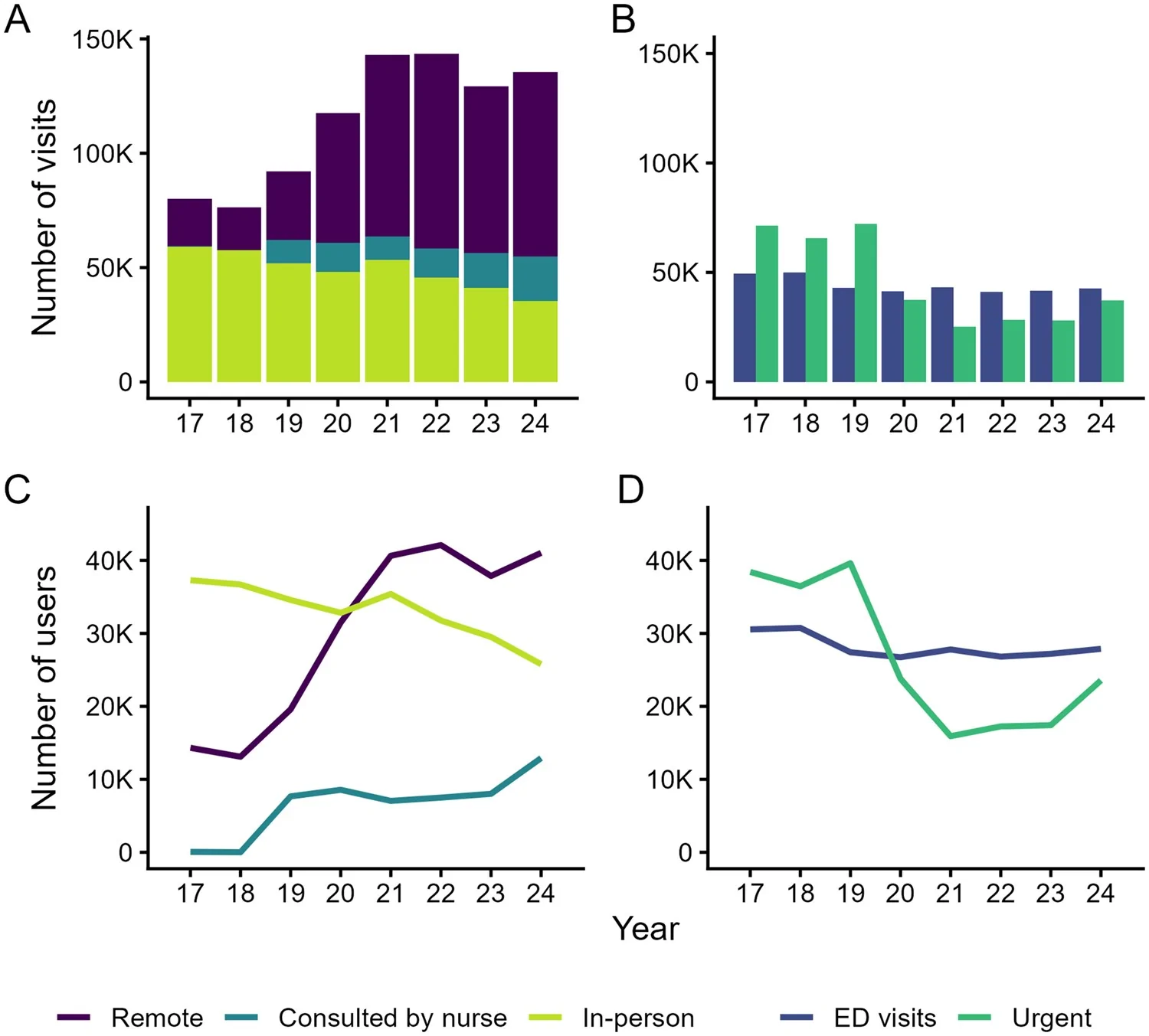
The number of different non-urgent GP consultations (a), the total number of urgent GP consultations and self-referred ED visits (b), and the respective numbers of patients accessing these services (c, d) in North Karelia in 2017–2024.

The quantitative results supported the lived experiences of patients and workforce: increase in remote care and decrease of in-person GP consultations. Over time the total number of all GP contacts in North Karelia slightly increased (from 151 268 in 2017 to 172 811 in 2024) while the total number of patients remained rather stable (61 948 and 64 232, respectively). However, the number of non-urgent in-person GP consultations decreased by 40%, while remote ones nearly quadrupled (Fig. 1). Though the total number of patients accessing non-urgent GP care increased 37% (from 41 106 to 56 122), the number of them with in-person GP consultations decreased by 31%. The number of urgent GP contacts (vast majority of them in-person consultations [data not shown]) halved during the COVID-19, but self-referred ED visits remained stable. Similar development with lower numbers emerged for the number of patients (Tables S1 and S2).

Over time the number of individual GPs providing at least one urgent or non-urgent consultation in North Karelia increased: from 348 in 2017 to 589 in 2024. Of these, the proportions of those who provided consultations only in maximum of 5 days were, respectively, 25% and 43%. The estimated number of FTE GPs providing in-person consultations decreased by 26% (from 88 to 65), while the respective number of those providing any contact types increased 25% (from 91 to 114). This increase seemed to mainly occur due to transition towards remote care: the estimated number of FTE GPs providing remote consultations increased 84% (from 44 to 80) (Tables S3 and S4).

**Figure 2.**
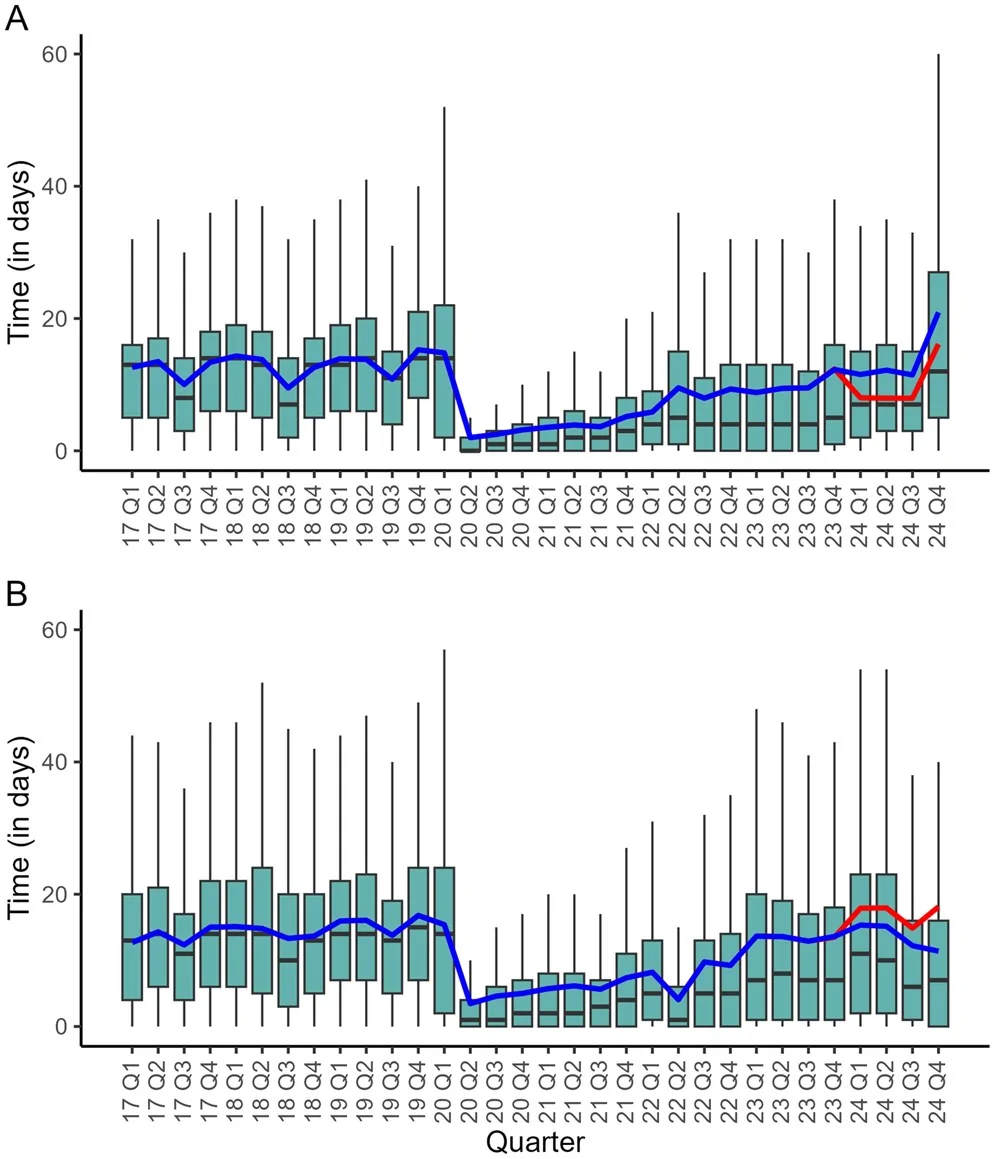
Time from care needs assessment by nurse (a)—or from initial contact to PC when no care needs assessment or excluded from the statistics (b)—to non-urgent in-person GP consultations in North Karelia in 2017–2024.

The waiting times for non-urgent in-person GP consultations remained stable before the COVID-19, dropped during the COVID-19 and increased afterwards (Fig. 2). The updated waiting-time guarantee legislation introduced a new definition for calculating these waiting times, which seemingly reduced them. However, no improvement emerged when calculating these statistics with old definition.

**Figure 3.**
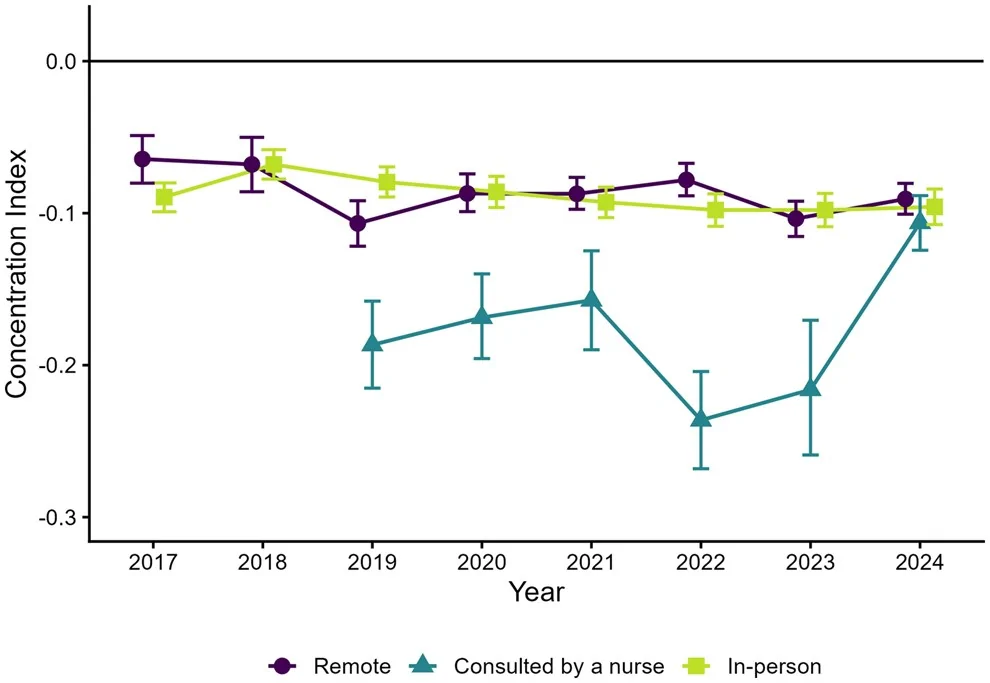
Over time development of concentration indices of non-urgent GP consultations by income quintiles in North Karelia in 2017–2024.

Over time development of Cs showed that provision of in-person and remote GP consultations slightly favoured those with lower incomes—i.e. their socioeconomic distribution was unaffected by the rapid shift to remote care (Fig. 3).

Sensitivity analyses showed similar findings when restricted to the municipality from which the qualitative data were collected, although the increase in remote GP consultations over time and the proportion of GPs working a maximum of 5 days were even more pronounced [data not shown].

## Discussion

This mixed-methods study offers important insights into PC users’ lived experiences after a policy update that aimed at improving PC access, with a prevailing narrative that included consequent reduction of national waiting times. A clear conflict emerged between lived experiences and national statistics. To better understand the qualitative findings, we analysed register data and focused on waiting times to PC, an area that remains less studied than waiting times to specialized care [21, 22].

We found mixed evidence on how the update of the Finnish waiting-time guarantee legislation impacted PC access in rural North Karelia. Patients and workforce reported increasing barriers in accessing GP care due to long-standing underinvestment in public PC and regional GP shortages, and especially older people emphasized challenges in navigating increasingly remote care. In 2017–24, the focus of GP work shifted rapidly from in-person consultations to remote ones. Some patients were able to benefit of this change: public PC reached further patients with no changes to pro-poor distribution of GP consultations or to usage of hospital emergency. Waiting time statistics missed these phenomena and its improvement seemed mere artefact of a revisited waiting time definition. However, to reach the stricter requirements, PC providers seem to have elevated criteria for accessing GP care.

Our findings highlight that the success of a health policy change should not be assessed solely based on single statistics. Overall, the validity and usefulness of the waiting time registries have been questioned as they are prone to technical errors, different interpretations and intentional manipulation of data [23]. For example, the Finnish waiting time statistics are often misleadingly generalized to mirror the whole PC access, even though they are calculated from very precisely defined initial consultations which account for no more than 40% of all non-urgent GP consultations [11]. Moreover, significant differences remain in how waiting times are measured and collected internationally [24]. To promote international comparability, countries should agree on common definitions or at least indicators for measuring waiting times [25].

Future research should continue to widen the scope of how access is defined and measured and more broadly, how policies aimed at increasing PC access are evaluated [7, 8, 26]. Attention should also be paid to the content of provided care and unwanted outcomes. Mixed-methods studies that combine qualitative and quantitative approaches can enrich both the evidence base and the evaluation of health policies. Lessons learned from Finland can provide other countries clues on how to elevate both patient and workforce perspectives in the design, implementation, and evaluation of health policies, improving their ‘human fit’ [12].

Waiting times to PC are less often a policy issue than those to elective treatments, and only a few countries, such as Finland, has set maximum waiting times for PC providers [27, 28]. The changes in the Finnish waiting-time guarantee legislation also illustrate how political determinants shape health systems [29–31]. The initial update of waiting-time guarantee included additional resources for strengthening long under-resourced public PC, but these resources were removed when a new government dismantled the initial goals to cut costs. In fact, austerity policies have increasingly steered public healthcare in Finland since the structural reform in 2023 [32, 33]. This change has occurred even though austerity policies risk complicating healthcare access especially for vulnerable groups [34, 35], and without clear political consensus, which should be one of the key prerequisites for strengthening health systems [36]. Overall, we argue that these back-and-forth policy decisions in Finland (i) exemplify the lack of consistent political commitment to strengthen public PC, and (ii) may have accelerated the shift towards a more remote PC system and elevated the criteria for accessing GP care.

### Strengths and limitations

The main strength of this study was its mixed-methods approach that enabled us to combine the detailed perspective obtained from patients’ and healthcare workforce with a broader one obtained from comprehensive and linked individual-level register data.

Though there are no published validation studies for public PC consultations in Register for Primary Health Care Visits, its data coverage is continuously monitored with no known gaps for North Karelia [11, 37]. Overall, the Finnish register data is considered of good quality [38, 39]. Our measure of FTE GPs—estimated from consultations and average working times—would have benefited of actual workforce data. The administrative structure of North Karelia WBSC was established in 2017 as a pilot for the large-scale health and social care reform–and was thus unaffected by the reform implementation in 2023 [40]. The COVID pandemic strongly impacted PC provision, which we addressed by over time analyses.

The qualitative interviews were of cross-sectional nature with participants consisting only of PC users, mainly older adults. Sensitivity analyses of the quantitative data indicate that the qualitative findings may reflect a health care centre where the transition towards telemedicine has progressed slightly further than elsewhere in the region. Long-term qualitative data collection and quantitative focus on adverse events of PC non-users is needed in the future. Qualitative data are subject to research interpretation and potential coder bias during the thematic analysis. To address this, the analysis involved multiple rounds of coding and memo-writing, systematic engagement with the literature, and the presentation of preliminary qualitative findings to the wider research group throughout the process.

## Supplementary Material

ckag121_Supplementary_Data

## Data Availability

The qualitative data underlying this article cannot be shared publicly due to the privacy of individuals who participated in the study. The quantitative register data include sensitive individual-level information and cannot be shared publicly due to national data protection legislation and the General Data Protection Regulation. Permissions to access Finnish register data for research purposes can be obtained from the Health and Social Data Permit Authority Findata (https://www.findata.fi/en/services/data-requests/). Key points Lived experiences of patients and health workforce conflicted with improving waiting time statistics in the rural North Karelia after the update of waiting-time guarantee legislation. Interviews and observations highlighted increasing challenges in access to care, rapid shift towards telemedicine, stricter gatekeeping to GP consultations and long-time underinvestment in primary care. Register-based analysis corroborated the increase in remote care, questioned the improvement in waiting time statistics but also found that public primary care reached further patients with no changes to pro-poor distribution of GP consultations. Our findings highlight the importance of incorporating patient and workforce perspectives into the design and evaluation of access policies in general practice. Mixed-methods studies that combine qualitative and quantitative approaches can enrich both the evidence base and the evaluation of health policies.
