# Supplementary material for: Exploring primary care access in Finland: a mixed-methods study in the context of the updated waiting-time guarantee
Source: Eur J Public Health. 2026 Jul 14;36(4):ckag121. doi: 10.1093/eurpub/ckag121 (PMC13368859; doi:10.1093/eurpub/ckag121)
Supplement: ckag121_Supplementary_Data [file ckag121_supplementary_data.docx]

**Supplementary Table S1:** Annual numbers of general practitioner consultations and patients in public primary care as well as visits and patients in emergency department in North Karelia, Finland, 2017–2024.

| **Year** | **Number of consultations/ visits** | | | **Number of patients** | | |
| --- | --- | --- | --- | --- | --- | --- |
|  | Primary care total | Primary care non-urgent | Emergency department | Primary care total | Primary care non-urgent | Emergency department |
| 2017 | 151 268 | 80 012 | 49 538 | 61 948 | 41 106 | 30 547 |
| 2018 | 141 900* | 76 230* | 50 018 | 60 313 | 40 379 | 30 754 |
| 2019 | 164 285 | 92 183 | 42 899 | 64 780 | 45 556 | 27 413 |
| 2020 | 155 119 | 117 661 | 41 344 | 61 041 | 51 713 | 26 734 |
| 2021 | 168 260 | 143 093 | 43 284 | 64 964 | 59 085 | 27 796 |
| 2022 | 172 044 | 143 599 | 41 219 | 65 108 | 58 590 | 26 812 |
| 2023 | 157 178 | 129 158 | 41 733 | 61 881 | 55 064 | 27 201 |
| 2024 | 172 811 | 135 557 | 42 618 | 64 232 | 56 122 | 27 881 |

*Values rounded to prevent identification of small cells in Table S2

**Supplementary Table S2:** Annual numbers of general practitioner (GP) consultations and patients by the type and urgency of consultation in public primary care of North Karelia, 2017–2024.

| **Year** | **Number of consultations** | | **Number of patients** | |
| --- | --- | --- | --- | --- |
|  | Non-urgent | Urgent | Non-urgent | Urgent |
| *In-person GP consultations* | | | | |
| 2017 | 59 164 | 70 778 | 37 295 | 38 280 |
| 2018 | 57 516 | 65 275 | 36 693 | 36 348 |
| 2019 | 51 801 | 69 637 | 34 579 | 38 644 |
| 2020 | 48 144 | 32 869 | 32 823 | 21 552 |
| 2021 | 53 283 | 21 639 | 35 401 | 13 980 |
| 2022 | 45 680 | 25 632 | 31 768 | 16 009 |
| 2023 | 41 073 | 25 537 | 29 514 | 16 133 |
| 2024 | 35 455 | 26 464 | 25 778 | 17 834 |
| *Remote GP consultations* | | | | |
| 2017 | 20 781 | 458 | 14 317 | 441 |
| 2018 | 18 701 | 395 | 13 099 | 386 |
| 2019 | 29 969 | 1369 | 19 588 | 1301 |
| 2020 | 56 870 | 3349 | 31 500 | 2986 |
| 2021 | 79 605 | 2337 | 40 638 | 2083 |
| 2022 | 85 232 | 1850 | 42 101 | 1637 |
| 2023 | 72 842 | 1495 | 37 869 | 1399 |
| 2024 | 80 710 | 8356 | 41 038 | 6403 |
| *GP consulted by a nurse* | | | | |
| 2017 | 67 | 20 | 56 | 19 |
| 2018 | 10 | <3 | 10 | <3 |
| 2019 | 10 413 | 1096 | 7664 | 1046 |
| 2020 | 12 647 | 1240 | 8563 | 1143 |
| 2021 | 10 205 | 1191 | 7046 | 931 |
| 2022 | 12 687 | 963 | 7495 | 798 |
| 2023 | 15 243 | 988 | 8013 | 843 |
| 2024 | 19 392 | 2434 | 12 914 | 2169 |

**Supplementary Table S3:** Annual numbers of general practitioners providing consultations and of those who provided consultations for a maximum of 5 days in public primary care of North Karelia, 2017–2024.

| **Year** | **Total (n)** | **Max 5 days (n)** | **Max 5 days (%)** |
| --- | --- | --- | --- |
| 2017 | 348 | 86 | 24.7 |
| 2018 | 337 | 87 | 25.8 |
| 2019 | 342 | 106 | 31.0 |
| 2020 | 432 | 167 | 38.7 |
| 2021 | 555 | 196 | 35.3 |
| 2022 | 636 | 256 | 40.3 |
| 2023 | 667 | 270 | 40.5 |
| 2024 | 589 | 254 | 43.1 |

**Supplementary Table S4:** Annual numbers of full-time equivalent general practitioners (FTE GPs) providing consultations in public primary care in North Karelia, Finland, 2017–2024. Average working hours were divided by the average length of a GP working day (7h 47min)*.

| **Year** | **Average working hours/year**** | **Number of days individual GPs provided consultations** | | | **Estimated number of FTE GPs** | | | |  |
| --- | --- | --- | --- | --- | --- | --- | --- | --- | --- |
|  |  | Total | In-person | Remote | | Total | In-person | Remote | |
| 2017 | 1550 | 18 206 | 17 574 | 8672 | | 91.4 | 88.2 | 43.5 | |
| 2018 | 1560 | 18 925 | 18 241 | 8455 | | 94.4 | 91.0 | 42.2 | |
| 2019 | 1534 | 19 120 | 17 198 | 10 472 | | 97.0 | 87.2 | 53.1 | |
| 2020 | 1541 | 20 880 | 16 589 | 16 082 | | 105.4 | 83.8 | 81.2 | |
| 2021 | 1525 | 23 116 | 16 068 | 18 250 | | 117.9 | 82.0 | 93.1 | |
| 2022 | 1465 | 24 009 | 13 924 | 17 858 | | 127.5 | 73.9 | 94.8 | |
| 2023 | 1453 | 24 271 | 13 630 | 15 864 | | 130.0 | 73.0 | 84.9 | |
| 2024 | 1485 | 21 759 | 12 457 | 15 275 | | 114.0 | 65.3 | 80.0 | |

*Reference: Finnish Medical Association. *Työaika ja työhyvinvointi: Lääkärin työolot ja terveys [in Finnish]*. <https://www.laakariliitto.fi/wp-content/uploads/2025/01/Tyoolot-ja-terveys-2024.pdf> (26 January 2026, date last accessed).

**Reference: Statistics Finland. *Annual hours actually worked per employed and employee, by employer sector and industry (TOL 2008), persons aged 15 to 74*. <https://pxdata.stat.fi/PXWeb/pxweb/fi/StatFin/StatFin__tyti/statfin_tyti_pxt_13at.px> (15 January 2026, date last accessed).
